# eCBT Versus Standard Individual CBT for Paediatric Obsessive–Compulsive Disorder

**DOI:** 10.1007/s10578-022-01350-7

**Published:** 2022-04-22

**Authors:** Lucía Babiano-Espinosa, Gudmundur Skarphedinsson, Bernhard Weidle, Lidewij H. Wolters, Scott Compton, Tord Ivarsson, Norbert Skokauskas

**Affiliations:** 1 Regional Centre for Child and Youth Mental Health and Child Welfare, Department of Mental Health, Norwegian University of Science and Technology, Klostergata 46, 7030 Trondheim, Norway; 2 Faculty of Psychology, University of Iceland, Nyi-Gardur, Saemundargata 12, 102 Reykjavík, Iceland; 3 Department of Child and Adolescent Psychiatry, St. Olav’s University Hospital, Trondheim, Norway; 4 Department of Clinical Psychology and Experimental Psychopathology, University of Groningen, Groningen, The Netherlands; 5 Accare Child Study Center, Groningen, The Netherlands; 6 Department of Psychiatry and Behavioral Sciences, Duke University School of Medicine, Durham, NC USA; 7 Institute of Neuroscience and Physiology, Sahlgrenska Academy, University of Gothenburg, Gothenburg, Sweden

**Keywords:** Obsessive–compulsive disorder (OCD), Cognitive behavioral therapy (CBT), Enhanced cognitive behavioral therapy (eCBT), Children, Adolescents

## Abstract

Obsessive–compulsive disorder (OCD) is characterized by recurring obsessions and compulsions often with severe impairment affecting 1–3% of children and adolescents. Cognitive behavioural therapy (CBT) is the therapeutic golden standard for paediatric OCD. However, face-to-face CBT is limited by accessibility, availability, and quality of delivery. Enhanced CBT (eCBT) a combination of face-to-face sessions at the clinic and treatment at home via webcam and a supportive app system aims to address some of these barriers. In this pilot study, we compared eCBT outcomes of 25 paediatric patients with OCD benchmarked against traditional face-to-face CBT (n = 269) from the Nordic Long-term OCD Treatment Study, the largest paediatric OCD CBT study to date. Pairwise comparisons showed no difference between eCBT and NordLOTS treatment outcomes. Mean estimate difference was 2.5 in favour of eCBT (95% CI − 0.3 to 5.3). eCBT compared to NordLOTS showed no significant differences between response and remission rates, suggesting similar effectiveness.

## Background

Obsessive–compulsive disorder (OCD), characterized by obsessions and/or compulsions affects 1–3% of children and adolescents [1, 2]. Obsessions include recurrent disturbing thoughts, images, or impulses, and compulsions include repeated behaviours and mental acts, performed to alleviate stress or anxiety [3]. Without treatment, the disorder often leads to functional impairment [4, 5] and reduced quality of life [6]. The gold standard treatment for OCD is cognitive behavioural therapy (CBT) with exposure and response prevention (ERP). Selective serotonin reuptake inhibitors (SSRIs) might be added, if deemed necessary [7]. Doubts have been raised about the additional effect of medication [8–10]. CBT as monotherapy led to clinical remission in 39% of the patients in the Pediatric Obsessive–Compulsive Disorder Treatment Study (POTS) [11], and in 49.4% of patients among those who finished the treatment in the Nordic Long-term OCD Treatment Study (NordLOTS), the largest CBT study for paediatric OCD to date [12]. Not all children benefited substantially from the treatment: for example, 66 of 241 (27.4%) patients who completed treatment were classified as non-responders in the NordLOTS [9, 12]. In addition, there are still organizational, ideological, and practical barriers to treatment that limit the availability of CBT and its accessibility for some patients. For example, concerns have been raised about the shortage of experienced therapists, especially in remote areas [13]. There have been previous initiatives attempting to address at least some of the barriers to treatment by using internet CBT. For instance, Storch et al. [14] treated 31 patients in 12 weekly sessions delivered through video teleconferencing (VTC). Findings showed 56.1% reduction of the CY-BOCS total score after 14 sessions of webcam-delivered CBT. Farrell et al. [15] evaluated an intensive treatment consisting of 3 face-to-face CBT sessions, followed by a 3-week maintenance program via webcam for 10 children and youth, using a multiple baseline controlled design. The results showed 49% reduction in the CY-BOCS total score.

With the overall goal of improving access and outcomes, we developed an innovative treatment package for children and adolescents with OCD by integrating internet technology with well-validated principles of CBT. The enhanced CBT (eCBT) manual is derived from the NordLOTS manual and a Dutch CBT manual for paediatric OCD [16, 17]. The eCBT intervention is an innovative treatment package for children and adolescents with OCD. It includes five components which are integrated in the eCBT treatment process: VTC sessions, face-to-face sessions, an app system aimed to support the treatment structure with a psychoeducation tool, reminders and descriptions of exposure exercises, supportive messages, and frequent online ratings which allow direct feedback to the patient. The app system consists of a smartphone app for children and for parents, and a web-based application for therapists on the computer, which are all interconnected. The app provides information about OCD and CBT (psychoeducation videos), supports and structures ERP exercises at home, and closely monitors treatment progress. The eCBT package has been described in detail elsewhere [18]. eCBT aims to enhance traditional CBT (i.e., as delivered in the NordLOTS) by combining face-to-face sessions at the clinic and treatment at home via webcam, and a supportive app system, to facilitate transfer of exposure exercises from the therapist’s office to the home environment, improving implementation of ERP in daily life. The main goal of eCBT is to improve treatment satisfaction and compliance, to intensify exposure and response prevention, and ultimately to improve treatment outcomes. Both motivation and good technique is crucial for exposure work. In addition, homework completion is an important component of ERP. A review of homework completion in ERP [19] concluded that future work on technology use with ERP homework is warranted. Children often show most commitment to their homework in the first days after a treatment session, and then efforts gradually decrease. Scheduled phone calls with the therapist appeared to improve homework completion in a study with adults [20]. A combination of face-to-face sessions with in-between webcam sessions at home offers a possibility to follow up and to carry out therapist-assisted exposure exercises in the patient’s home. This may increase motivation and improve exposure technique, treatment intensity, and homework completion. The app offers a scaffolding construction for the treatment, while making use of children’s and adolescents’ affinity with internet technology. For patients and parents, this higher treatment intensity is achieved with less effort, because the number of face-to-face sessions demanding transport, parking, time off from school for the children and from work for the parents was reduced, and partly replaced by webcam sessions at home. A preliminary evaluation of acceptability and feasibility suggested that eCBT was highly accepted and feasible. Children’s and parents’ feedback on the Client Satisfaction Questionnaire-8 yielded mean scores of 27.58 (SD 0.67) for children and 29.5 (SD 3.74) for parents (range 8–32) and none of the participants dropped out from treatment [21].

The aim of the present study is to evaluate the effectiveness of eCBT, an Internet-enhanced version of the earlier NordLOTS CBT. For this reason, we compared eCBT with NordLOTS CBT outcomes in a pilot open trial benchmarked against NordLOTS as a first step. The NordLOTS established effectiveness and feasibility of CBT for OCD in the context of the Scandinavian healthcare system, including both specialized OCD clinics and general child psychiatric outpatient clinics [22, 23]. Because of the similarities in assessment and sample characteristics, we used the established NordLOTS outcome standards as a reference frame for comparison with eCBT outcomes. Our hypothesis is that the eCBT efficacy results are noninferior to results found in the NordLOTS.

## Methods

### Participants

**Fig. 1:**
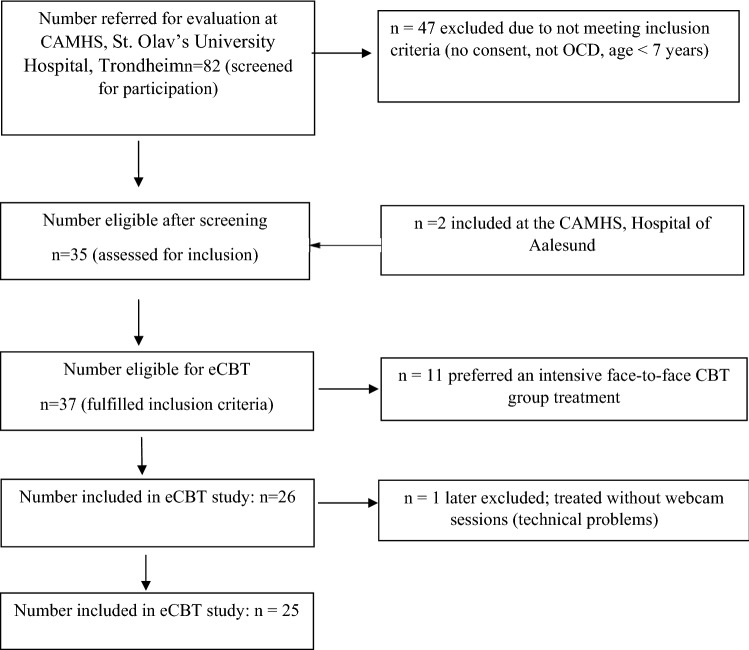
Patient recruitment flowchart

Inclusion criteria were identical in both studies, with the exception that patients with OCD and comorbid autism spectrum disorder (ASD) were not excluded from eCBT study. For an overview of inclusion and exclusion criteria for eCBT and NordLOTS see Table 1. The patient recruitment process is detailed in a flowchart (Fig. 1).

**Table 1:** Inclusion and Exclusion criteria for eCBT and NordLOTS studies

eCBT	NordLOTS
*Inclusion criteria*	
Primary DSM-5 diagnosis of OCD	Primary DSM-IV diagnosis of OCD
CY-BOCS entry score of 16 or above	CY-BOCS entry score of 16 or above
7–17 years of age	7–17 years of age
Patients with OCD, ASD, and ADHD eligible	Patients with OCD and ADHD eligible, after having been stabilized on medication for at least 3 months prior to entry
*Exclusion criteria*	
Presence of other psychiatric disorders (according to the DSM-5) with higher treatment priority (i.e., primary anorexia nervosa, psychosis, severe depression)	Presence of other psychiatric disorders (according to the DSM-5) with higher treatment priority (i.e., primary anorexia nervosa, psychosis, severe depression)
Intellectual disability	Intellectual disability ASD (PDD-NOS was allowed if OCD symptoms were most impairing)
Ongoing psychological treatment for OCD	A previous trial of exposure-based CBT for OCD less than 6 months prior to inclusion
	Medication treatment with an SSRI less than 6 months prior to inclusion
Inadequate language proficiency by the patient or the parent	Inadequate language proficiency by the patient or the parent

#### eCBT

The eCBT study was conducted in child and adolescent mental health services (CAMHS), St. Olav’s University Hospital, Trondheim, Norway (n = 23) and CAMHS, Hospital of Aalesund, Norway (n = 2). A total of 82 children and adolescents with suspected OCD were referred to CAMHS, St. Olav’s University Hospital, Trondheim between January 2018 and November 2019 and evaluated for participation in the study. Of these, 47 did not fulfil inclusion criteria. Twenty-six of the 37 eligible patients (70%) were informed about the eCBT study and agreed to participate, 11 (30%) preferred an intensive face-to-face CBT group treatment. The first patient included in the study did not have webcam sessions due to technical problems and was excluded from analyses (Fig. 1). As a result, the final sample consisted of 25 participants. All twenty-five participants completed the treatment. Mean age of participants (N = 25) was 13.1 years (SD 2.9), with slightly more female (56%) than male participants, and 97% having a Scandinavian background.

#### NordLOTS

A total of 269 children and adolescents, recruited at five main study sites in Denmark, Sweden, and Norway between September 2008 and June 2012, were included in the NordLOTS. Assessment and treatment were performed in 18 public community mental health clinics with general referrals and at two specialized OCD clinics (Aarhus, Denmark, and Gothenburg, Sweden) [12].

Participants’ characteristics are reported in Table 2. At baseline, the mean age of the NordLOTS participants was 12.8 years (SD 2.7 years, range 7–17), and 51.3% were girls. Ethnicity was primarily Scandinavian: 97% of the participants had one or both parents of Scandinavian origin.

**Table 2:** Patient characteristics

Variable	NordLOTS	eCBT	*p*-value
n	269	25	–
Age, year, mean (SD)	12.8 (2.7)	13.1 (2.9)	0.598
Age of OCD onset, year (SD)	11.7 (3.0)	9.7 (3.7)	**0.002**
Female participants, n (%)	138 (51.3)	14 (56.0)	0.653
Male participants, n (%)	131 (48.7)	11 (44.0)	
Family status, n (%)			
Parents living together, n (%)	173 (64.3)	21 (84.0)	**0.047**
Other, n (%)	95 (35.3)	4 (16.0)	
CY-BOCS, mean (SD)			
Total score at baseline	24.6 (5.1)	25.8 (5.4)	
Obsession score at baseline	12.3 (2.8)	13.1 (2.7)	0.147
Compulsion score at baseline	12.3 (2.7)	12.6 (3.1)	0.553
Comorbid disorders at baseline (K-SADS PL)			
Any anxiety disorder, n (%)	52 (19.3)	6 (24.0)	0.572
Any depressive disorder, n (%)	10 (3.7)	1 (4.0)	0.939
ADHD, n (%)	24 (8.9)	3 (12.0)	0.608
ODD/CD, n (%)	10 (3.7)	0 (0)	–
Tic disorder, n (%)	49 (18.2)	4 (16.0)	0.785
ASD, n (%)	1 (0.4)	4 (16.0)	** < 0.001**
Encopresis, n (%)	0 (0)	1 (4.0)	–
PTSD, n (%)	1 (0.4)	2 (8.0)	** < 0.001**
Eating disorder, n (%)	0 (0)	2 (8.0)	–
Number of co-occurring diagnoses, n (%)			0.072
None	163 (62.8)	13 (52.0)	
1	62 (23.0)	8 (32.0)	
2	25 (9.3)	2 (8.0)	
≥ 3	13 (4.9)	2 (8.0)	

Significant differences (p < 0.05) are indicated with boldface

*ADHD* attention deficit/hyperactivity disorder, *CD* conduct disorder, *CY-BOCS* Children’s Yale-Brown Obsessive–Compulsive Scale, *K-SADS-PL* Kiddie schedule for affective disorders and schizophrenia—present and lifetime version, *OCD* obsessive–compulsive disorder, *ODD* oppositional defiant disorder, *NA* non-applicable

In the NordLOTS, 269 patients were offered CBT, and 241 (89.6%) completed the treatment.

### Treatment

#### NordLOTS

NordLOTS treatment consisted of 14 sessions of weekly individual manualized CBT with ERP, based on the study protocol of March et al. [24] and modified by adding more extensive family participation [25] and adapted to fit Nordic cultural conditions [17]. The first sessions of the treatment included psychoeducation about OCD and treatment strategies and socializing to the treatment model of gradual exposure to threatening situations. For more detailed information about the NordLOTS treatment, see [12, 26, 27].

#### eCBT

The basic content of the eCBT treatment manual was comparable with the manual used in the NordLOTS. Like the NordLOTS, eCBT contains psychoeducation, ERP, cognitive interventions, and relapse prevention, with the focus on ERP.

In eCBT, 5 closely linked components are integrated: webcam sessions in combination with face-to-face sessions, an app system, a psychoeducation tool, and frequent online ratings with direct feedback to the patient. Another difference is the distribution of treatment sessions over time: the eCBT treatment protocol consisted of up to 10 weekly face-to-face sessions, combined with up to 12 shorter webcam sessions, delivered over a 14-week period. In eCBT, parents were involved by default. For a more detailed description of the eCBT treatment manual, see Wolters et al. [18].

### Measures

The same measures were used in both the eCBT study and NordLOTS.

The Schedule for Affective Disorders and Schizophrenia—Present and Lifetime version (K-SADS-PL) is a widely used standardized diagnostic interview for the assessment of psychiatric disorders in children and adolescents [28]. The instrument was used both to confirm OCD diagnoses and to evaluate the presence of comorbidity. The K-SADS-PL has excellent inter-rater reliability [29] and validity [30, 31].

The Children’s Yale-Brown Obsessive–Compulsive Scale (CY-BOCS) [32] is a semi-structured interview developed for the assessment of OCD symptoms in children and adolescents. The instrument consists of a subscale for obsessions and another for compulsions, scored by the clinician, based on interviews with each child and parent or caregiver informant. The CY-BOCS total score (range 0–40) is the sum of the subscale scores (range 0–20) for obsessions and compulsions. The CY-BOCS showed reasonable reliability and validity, high internal consistency (α = 0.90) and test–retest reliability (ICC  0.79 for the total score), and good inter-rater agreement (ICC  0.84 for the total score) [33, 34].

### Statistical Analysis

Participants with missing and non-missing data were compared by baseline CY-BOCS total score, sex, age, age of onset, and comorbidity. None of these comparisons showed significant differences at baseline, and no differences were found between participants with and without missing data. For that reason, we considered missing data as missing at random.

We computed the percentage of patients with OCD symptoms in the clinical range (CY-BOCS ≥ 16) and in remission (remission defined as CY-BOCS ≤ 12), and the percentage of treatment responders (treatment response defined as ≥ 35% symptom reduction on the CY-BOCS plus CGI-I (Clinical Global Impressions scale—Improvement’) rating of 1 or 2 “(very) much improved”). Effect size (d) was calculated by the mean difference CY-BOCS score pre- versus post-eCBT, divided by the standard deviation of the difference score pre- versus- post-eCBT.

We used linear mixed models (LMMs) to evaluate outcome changes on the CY-BOCS total score [35, 36]. The model included all available data to estimate model parameters. LMMs are commonly recommended both for superiority and noninferiority analyses because of their ability to handle missing data and correlated and repeatedly measured observations [37]. We used restrictive maximum likelihood estimation and included fixed effects for time (baseline and week 14), treatment cohort (NordLOTS vs. eCBT), and their interaction. The model included random effects for intercept. An unstructured covariance was used to account for correlated observations. For benchmarking against NordLOTS, we explored noninferiority following current guidelines [38]. An important part of a noninferiority calculation is to examine the inferiority margin [38]. We determined whether eCBT was inferior to the NordLOTS results by using the confidence interval (CI). If the lower limit of the two-sided 95% CI of the difference of the mean is less than the noninferiority margin (pre-specified), we can establish noninferiority [37, 38]. We set noninferiority at 4 points on the CY-BOCS scale using a 95% CI. eCBT would be non-inferior to the NordLOTS CBT if the upper limit of the 95% CI for the difference between eCBT and the NordLOTS CBT were less than 4. This means that we would be 95% confident that the real value of the difference between these cohorts (at week 14) was not worse than 4 points on the CY-BOCS total score.

Multivariate χ^2^ tests were conducted on binary outcomes (e.g., remission). Multiple imputation was used to replace missing data. This was done with a sequential regression multivariate imputation algorithm [39]. This imputation model included all baseline demographics and outcome measures, and a total of 20 multiple imputations were generated according to guidelines [40, 41]. The SAS 9.4 proc mi was used to generate the 20 imputation datasets. Outcomes reported were calculated using Rubin’s rules [39] to combine the results of the 20 identical analyses, using the SAS 9.4 proc MIANALYZE procedure. This was done on each of the 20 imputed datasets, and the results were combined and reported as an F statistic. Tests were two-tailed, and a p-value of less than 0.05 was considered to indicate statistical significance. The computation of a combined F statistic was conducted with the SAS macro COMBCHI [42]. We used SAS statistical software, version 9.4 to conduct the LME (using the proc mixed procedure) and multiple imputation. All other analyses were performed using SPSS version 26.0.

### Calculation of Sample Size

To explore noninferiority in this pilot open trial benchmarked against NordLOTS, power calculations indicate that 4 points on the CY-BOCS total score means that 21 participants in the eCBT group would be sufficient to show noninferiority, with alpha = 0.05 and beta 0.20.

## Results

### Patient Characteristics

At baseline, the mean CY-BOCS total score was 24.6 (SD 5.1) for NordLOTS and 25.8 (SD 5.4) for eCBT (Table 2). Statistically significant differences between groups were present for age of onset of OCD, with mean age of OCD onset 11.7 years (SD 3.00) in the NordLOTS and M 9.7 (SD 3.7) in the eCBT sample t(291) = -3.131, p = 0.002). ASD t(291) = 32.697, p < 0.001, and PTSD t(291) = 12.718, p < 0.001 were more frequent in the eCBT sample. We found no statistical differences between groups for baseline CY-BOCS, family status, other comorbid disorders, or a number of co-occurring diagnoses at baseline. Two eCBT patients were receiving concurrent medication for non-OCD psychological disorders (guanfacine and risperidone). None of the patients received any medication for OCD. For two patients it was difficult to keep up webcam sessions due to comorbid problems, i.e., ASD and eating disorder, resulting in only one completed webcam session for each participant. Both patients received four extra face-to-face sessions to compensate for therapist time. Another patient received 11 face-to-face sessions instead of the protocolized 10 sessions, which included treatment of a comorbid specific phobia.

### Outcomes

**Fig. 2:**
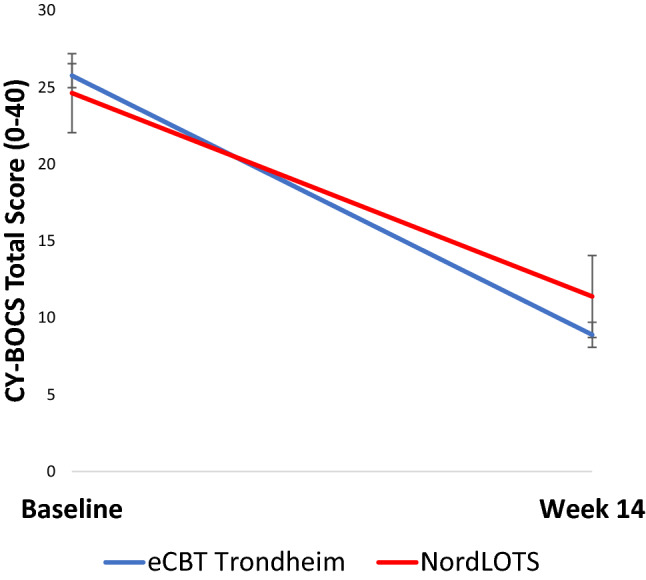
Adjusted intent-to-treat CY-BOCS total score across treatment cohorts

Adjusted intent-to-treat CY-BOCS total scores across treatment cohorts are shown in Fig. 2. Pairwise comparisons post-treatment showed that the difference between eCBT and NordLOTS treatment outcomes was not statistically significant [t(264) = 1.76, p = 0.08]. The mean estimate difference was 2.5 (in favour of eCBT), with the 95% CI between − 0.3 and 5.3. Further information on parameter estimates may be found in Table 3.

**Table 3:** Parameter estimates from fitting elevation and slope to the CY-BOCS

Parameter	Final model
Composite model, estimate (SE)	
Intercept	25.760 (1.304)***
Weeks	− 1.205 (0.121)***
Group	− 1.136 (1.363)
Week*group	0.259 (0.127)*
Variance components	
Level-1—within person	34.317 (2.929)***
Level-2—in initial status	8.174 (2.597)***

*p < 0.05, ***p < 0.001

The estimated change in mean CY-BOCS total scores from baseline to posttreatment was significantly greater in the eCBT group than in NordLOTS (change in mean difference = 3.6) [t(264) = 2.04, p = 0.04] (Table 4).

**Table 4:** Posttreatment group-specific outcomes and response rates

Primary outcomes	Estimated mean or rate (95% CI)^a^		Effect sizes (95% CI)^b c^
	eCBT	NordLOTS	
CY-BOCS total score	8.9 (6.2, 11.6)	11.4 (10.6, 12.2)	0.35 (− 0.06, 0.76)
Reduction from baseline CY-BOCS total score	− 16.9 (− 20.2,13.5)	− 13.2 (− 12.2, − 14.3)	0.41 (0.00, 0.82)
Symptoms in clinical range (CYBOCS ≥ 16)	24.0% (9.4%, 45.1%)	29.1% (23.3%, 34.8%)	0.11 (− 0.29, 0.52)
Remission (CY-BOCS ≤ 12)	68.0% (46.1%, 89.9%)	57.6% (51.4%, 63.8%)	0.21 (0.20, 0.62)
Response rate	68.0% (46.5%, 85.1%)	59.6% (53.5%, 65.8%)	0.17 (− 0.24, 0.58)
Attrition rate	1 (4.0%)	26 (10.7%)	

^a^For CY-BOCS total score, estimated mean score at posttreatment from the fitted LMM. For the categorical outcomes, the estimated rate at posttreatment

^b^For CY-BOCS total score, the between-groups difference in estimated mean score at posttreatment. For the categorical outcomes, between-groups difference in rate at posttreatment

^c^Positive effect size suggests that eCBT was more effective

A total of 59.6% of participants in the NordLOTS and 68.0% of participants in the eCBT cohort responded to treatment. The multivariate χ^2^ test showed no statistically significant differences between groups (p = 0.415).

We also examined differences in attrition and remission rates. Attrition rates were similar, with no significant differences between groups (χ^2^ (1, 293) = 0.880, p = 0.348). Remission rates were 68.0% for the eCBT group and 57.6% for the NordLOTS group. The multivariate χ^2^ test showed no statistically significant differences between the groups (p = 0.317).

Lastly, we examined differences in the number of participants with symptoms still within the clinical range (CY-BOCS total score 16 or above) at posttreatment. In the eCBT group 24.0% of participants were still within the clinical range, compared with 29.1% in the NordLOTS group. This difference was not significant (p = 0.593).

## Discussion

The aim of this study was to examine the effectiveness of eCBT by comparing the outcomes with historical outcomes of well-established traditional CBT for paediatric OCD. eCBT offers traditional CBT in an enhanced format, including additional webcam sessions for therapist-assisted exposure exercises at home and a supportive app system. As a first step in examining the efficacy of this newly developed protocol, we compared eCBT with standard CBT delivered face-to-face, by benchmarking the results against NordLOTS, the largest study to date on the effectiveness of CBT for paediatric OCD. The results show that eCBT is at least equally effective as the NordLOTS.

eCBT and NordLOTS samples were comparable in terms of OCD severity scores at baseline, male to female ratio, family setting, and rate of comorbidity. Differences existed in the inclusion of ASD in the eCBT study, while this was an exclusion criterion in the NordLOTS sample. However, Pervasive Development Disorder—Not Otherwise Specified (PDD-NOS) was allowed in the NordLOTS if symptoms of OCD were most impairing; hence there was one patient also with ASD in the NordLOTS sample. Four of the 25 participants included in the eCBT group had a diagnosis of ASD. In addition, age and age of OCD onset were slightly lower in the eCBT group than in the NordLOTS sample.

The mean estimated difference of CY-BOCS total scores − 2.5 (95% CI − 0.3 to − 5.3) was not statistically significant within the 4-point difference margin. The estimated change in mean CY-BOCS total scores from baseline to posttreatment was significantly greater in the eCBT group than in NordLOTS. This could indicate that eCBT might be more effective in reducing OCD symptoms than standard CBT. However, these results need to be considered preliminary, and need to be properly examined using a randomized controlled trial with head-to-head comparison. We found no significant differences between response and remission rates in the respective studies.

Attrition rates did not differ significantly between eCBT and NordLOTS (4.0% versus 10.7% respectively; p = 0.289), which indicates that eCBT offers a feasible format for most participants, comparable to standard CBT. As already mentioned, the eCBT sample included 4 participants (16.0%) with ASD. All four had large reductions in pre to post CY-BOCS scores (34/17, 21/0, 18/12 and 25/0). Treatment of children and adolescents with OCD and ASD has been associated with lower response rates [43]. A review of the effectiveness of CBT for individuals with ASD and comorbid OCD concluded that standard CBT needs to be modified to address special needs of children with ASD, for example increased structure in the sessions, visual aids and cues, and considerable parental involvement [44]. Although these results are very preliminary, the first impression is that eCBT could offer a suitable format for children with ASD and comorbid OCD. The very positive outcome of eCBT in a few patients with ASD and OCD could be a promising direction for future research. Hopefully, future studies will be able to determine if components of eCBT, such as facilitating individual adjustments, transfer from the office to the home environment, and the possibility of therapist-assisted exposure work via webcam at home, may make this treatment appealing for children with OCD and ASD.

It is relevant to compare the results for eCBT with results from other Internet-enhanced CBT programs for paediatric OCD. The results for eCBT with 65.5% reduction of CY-BOCS total score from baseline to posttreatment evaluation seem to be in line with other studies using Internet technology to deliver CBT for children with OCD [45]. For example reported the mentioned studies by Storch et al. [14] 56.1% reduction of CY-BOCS total score after webcam-delivered CBT and Farrell et al. [15] 49% reduction of CY-BOCS score after three face-to-face CBT sessions, followed by a 3-week maintenance program via webcam. Other studies applying various degrees of Internet technology to deliver CBT to children with OCD reported somewhat lower reductions in CY-BOCS scores after treatment [45].

eCBT may help increase access to treatment by reducing geographical barriers and facilitating expert treatment by providing part of the treatment at home via webcam sessions. Although eCBT is not fully remote, the fewer number of face-to-face sessions limits travelling time and costs and may make treatment more patient-friendly and easier accessible even when patients live in distance from a qualified therapist. Previous results suggested that eCBT offers an acceptable and feasible treatment format; no major barriers to treatment were reported by participants or therapists [21]. Another advantage of eCBT is that exposure exercises can be performed in a naturalistic environment where the symptoms are most prevalent (usually at home) and directly modelled and supervised by the therapist. Practising and implementing exposure in daily life is essential for a successful treatment. Adapting the treatment schedule to individual needs, including online support from the therapist when practising exposure at home, may have the potential to reduce treatment failure and dropouts.

eCBT was conceptualized and data were acquired prior to the COVID-19 pandemic when safe online treatment was less common. The COVID-19 pandemic has catalyzed digital transformation in all aspects of our society, including CAMHS. The pandemic crisis underlined the need for treatment without direct contact during critical times. In addition, delivering larger treatment parts via webcam at home offers both more convenient treatment as well as easier access to treatment, and more possibilities for offering therapist-assisted exposure exercises in the patient’s home environment.

### Strengths and Limitations

A strength of this study is that similar sample characteristics facilitate comparison between samples. Both samples included patients with comparable degrees of OCD severity and comorbid disorders. The same assessment methods, with independent evaluators and similar treatment strategies, were used in both studies. In addition, the treatment concept of eCBT is derived from NordLOTS CBT and a similar Dutch treatment protocol, and differences regarding treatment delivery were well defined.

Main limitations include a small eCBT sample size and the absence of a direct comparison group. These limitations derive from the concept of the eCBT study as a pilot feasibility trial whose aim was to explore preliminary effectiveness, before continuing with randomized and controlled studies which demand much larger resources.

In this paper we report on and compare acute eCBT outcomes posttreatment. NordLOTS documented that the treatment gains were durable over a follow-up period of 3 years [23]. Also, for eCBT follow-up, observations will be necessary to study whether the treatment gains remain stable over time.

## Summary

The present study explores the preliminary effectiveness of enhanced CBT (eCBT) for children and adolescents with OCD. eCBT is a combination of face-to-face sessions at the clinic and treatment at home via webcam and a supportive app system aimed to address limitations to standard face-to-face CBT as accessibility, availability, and quality of delivery. In this pilot open trial, we compared eCBT outcomes of 25 paediatric patients with OCD benchmarked against standard face-to-face CBT (n = 269) from the Nordic Long-term OCD Treatment Study, the largest paediatric OCD CBT study to date. eCBT compared to NordLOTS showed no significant differences between response and remission rates, suggesting similar effectiveness. The mean estimate difference was 2.5 in favour of eCBT (95% CI − 0.3 to 5.3). As we found a significantly higher mean reduction of CY-BOCS scores in the eCBT group, it may have the potential to be more effective in reducing OCD symptoms than standard CBT. However, this latter finding needs to be examined in randomized head-to-head comparisons between groups and on a larger scale.

## Data Availability

The data and materials are available from the corresponding author on reasonable request.
